# Fast Methods for Finding Multiple Effective Influencers in Real Networks

**DOI:** 10.6028/jres.125.036

**Published:** 2020-12-31

**Authors:** Fern Y. Hunt, Roldan Pozo

**Affiliations:** 1 National Institute of Standards and Technology, Gaithersburg, MD 20899, USA

**Keywords:** approximation algorithms, hitting time, information dynamics, Monte Carlo methods, networks, random walks

## Abstract

We present scalable first hitting time methods for finding a collection of nodes that enables the fastest time for the spread of consensus in a network. That is, given a graph *G* = (*V, E*) and a natural number *k*, these methods find *k* vertices in *G* that minimize the sum of hitting times (expected number of steps of random walks) from all remaining vertices. Although computationally challenging for general graphs, we exploited the characteristics of real networks and utilized Monte Carlo methods to construct fast approximation algorithms that yield near-optimal solutions.

## 1 Executive Summary

The underlying properties of complex natural, engineered, and social systems are often revealed by representing them as an interconnected network of nodes. Each node (sometimes referred to as an agent) interacts directly only with other nodes to which it has a direct connection in the network. The study of the resulting dynamics give insight into the collective behavior of the system as a whole. A problem of major interest in network science is the identification of a subgroup of nodes whose interactions with neighbors will cause information to spread most efficiently throughout the network. This problem has many applications, from identification of computers through whom viruses will most quickly spread throughout a communication network to understanding of the social hierarchies in groups of animals.

In this paper we discuss a simplified model of node interaction that is used in the study of both computer network control as well as the spread of innovation in social networks. We present a set of heuristic algorithms that identify effective influencers, i.e., nodes whose interactions produce the most rapid approach to consensus. These algorithms are tailored for use in large real-world networks with so-called heavy-tailed degree distributions. The execution speed suggests that our approach could be used to investigate networks with more complex node interactions.

## 2 Introduction

The identification of nodes in a network that enable the fastest spread of information is an important, if not fundamental, problem in network control and design. It is applicable to the optimal placement of sensors, the design of secure networks and the problem of control when network resources are limited. Our approach to this problem has its origins in the widely studied consensus models ([1] and references cited there) and the related models of opinion dynamics and spread of social innovation [2-5]. In these models each individual node exchanges information with its neighbors and consensus is reached when all nodes have the same information values. In a modification of the consensus model that highlights the dynamics of influence, a pre-selected set of nodes guides the evolution of the system to a consensus value that depends on the values of the set. The effectiveness of these nodes, called *leaders* or *influencers* depending on the application, can be measured by the speed at which consensus is reached. Given a fixed cardinality, we call a vertex set that maximizes the speed over all sets with the same cardinality, an *effective influencer* set.

In related work, the mechanisms of social contagion in networks such as epidemics [6, 7] or rumors have also been the subject of intense research and progress [7]. As in the study of consensus models, the focus of attention has been on identifying the topological characteristics of nodes that are so-called superspreaders using measures such as degree, k-core and non-backing centrality of a node. Despite this effort, the identification of subsets rather than individual spreaders remains a challenge. In this paper we consider a dramatically simpler model to be able to address this problem. The heuristic approaches discussed here are potentially relevant to such models of spread.

The consensus process is defined here in terms of a random walk process on the nodes of a network graph. At each time step information exchange occurs because a random walker situated at a node takes a step to a neighboring node as in the standard consensus model [8]. In this work, the effectiveness of a subset of nodes is measured by the maximum expected time it takes a random walker to reach the set for the first time (first hitting time) [9]. Thus, the subset of most effective influencers among all sets of some given cardinality minimizes this quantity. This is the same criteria of effectiveness as our original definition. The reason is that the rate of convergence to consensus for any fixed set is in fact also determined by the first hitting time to the set. The eigenvalue of the relevant matrix and the first hitting time are related. Thus the most effective influencer among all sets of some constrained cardinality minimizes this quantity. We pose an optimization problem for minimizing an equivalent quantity and then we will present an approximate solution that is suitable for graphs from real-world applications.

The paper is organized as follows: Section 3 discusses the notation and concepts for network graphs and random walks needed to describe the communication model and the Monte Carlo-based method for solving the optimization problem. Section 4 discusses the relationship between the rate of convergence to consensus and the expected first arrival time of a random walker associated with the averaging matrix. We then present the optimization problem for finding the subset of target nodes that minimizes the first hitting or arrival times. Our principal contribution, appearing in Sec. 5, is an algorithm for approximating the solution that is suitable for large scale networks. Validating the results of the algorithm and its heuristic modifications is quite challenging. Section 6 addresses the adequacy of the Monte Carlo approximation of the first arrival time and suggests a method for evaluating the results of the algorithm based on the use of Chebyshev's inequality ([10, 11] for recent applications). Examples showing implementation of the algorithm and characteristics of the graphs where it is most effective can be found in Sec. 7. We conclude with a discussion of directions for future work in Sec. 8.

## 3 Notation

Our paper concerns random walks on a graph, for which we use the following notation and concepts. An exposition of Markov chain theory can be found in [12], while random walks on graphs are discussed in [13, 14]. A graph is denoted by *𝒢* = (*V, ℰ*), with *V* representing the sets of nodes and *ℰ* representing its edges. An individual node is indicated by small roman letters, *e.g.*, *i, j*. The edges of the graph are assumed to be undirected and the graph itself connected. A subset of nodes *A* ⊆ *V*, has a complementary set *A^c^*= *V* \ *A*. For definitions we use the notation *A* ≐ *B* to denote ‘*A* defined as *B*’.

## 3.1 Random Walks

The position at any time of a random walker traversing the nodes of a graph in discrete time steps can be described by a random process known as a Markov chain. Here the states of the Markov chain are nodes of the graph and the state at any time is the node the random walk occupies at that time. Given an initial probability distribution on the nodes, the distribution after a single time step can be found in terms of the transition probability matrix *P*. Let *p* be the column vector of size |*V* |, for which the *i*th component *p_i_* is the probability that the Markov chain is in state *i*. In other words, a random walker is situated at node *i* at a given time step. The position at the next step is described by the probability distribution *p^T^ P*. The (*i, j*) th entry of *P*, *P*(*i, j*) is the conditional probability that the random walker steps to *j* given that the walker was at *i* in the previous time step. Notions of conditional expectation are also used. The notation for the expected or mean value of a random variable *X* is 𝔼[*X* ], while the expected value *X* given the random walker is initially at node *i* is 𝔼*_i_*[*X* ]. More generally if the initial position of the walker is defined by a probability distribution *µ*, then the expected value of *X* given *µ* is 𝔼*_µ_* [*X* ]. When the Markov chain is irreducible and aperiodic, the distribution of possible positions of the random walker after a long time is described by the so-called stationary distribution, denoted by the row vector *π^T^* . In addition we will assume the Markov chain is reversible so that *π*(*i*)*P*(*i, j*)=*π*(*j*)*P*(*j, i*). The most common example of such a process is the simple uniform random walk for which the transition probabilities are

$P\left(i,j\right)=\left\{\begin{array}{l} \frac{1}{\deg\left(i\right)},\text{ if }j⁓i;\\ 0\text{ otherwise.} \end{array}\right.$(1)

A node *j* is adjacent to *i* if and only if (*i, j*) or (*j, i*) ∈ *ℰ* Here the relation node *j* is adjacent to *i* is indicated by *j* ⁓ *i* and *deg*(*i*) is the total number of nodes adjacent to *i*. The results described here can apply to any walk on a directed graph described by a reversible Markov chain by allowing weighted edges, *i.e.*, a non-symmetric adjacency matrix. In this case the corresponding transition probability would measure the weighted strength of a node rather than its degree. Although this random walk is not aperiodic the so-called lazy random walk is aperiodic. The transition matrix of the lazy random walk is (*I* + *P*)*/*2, where *I* is the identity matrix. The mean first hitting time to sets is double that of the original random walk, so with this modification any result that requires the Markov chain to be aperiodic can apply to the periodic chain.

In Sec. 4 we will discuss the random variable that describes the first time a random walker visits a subset *A*, *i.e.*, the first hitting time of *A, T_A_≐*min{*t* ≥0: *X_t_* ∈ *A*}, where *X_t_* is the position of the walker at time *t*. We will also use the notation $T_{A}^{i}$, the first hitting time of *A*, given the random walk began at node *i*. The mean or expected value of $T_{A}^{i}$ is written as 𝔼[$T_{A}^{i}$] and because of this, the value is independent of the history of the walk before it was at *i*,𝔼[$T_{A}^{i}$] = 𝔼*_i_*[*T_A_*]. The dynamics of the random walk before time *T_A_* are described by the submatrix of *P* obtained by crossing out the rows and columns of *P* corresponding to the nodes of *A*. We denote this matrix by *P_A_*. The formal setting for the random walk is the triple (Ω, *ℬ,* ℙ) where Ω, the sample space of the Markov chain, is the set of nodes *V*, *ℬ* is the corresponding class of events or sets of outcomes associated with random walk paths and ℙ is a probability measure on random walk paths induced by the transition probability. Since the algorithm we present in Sec. 4 involves a Monte Carlo sample approximation we take the time to present the notation used to describe this approximation.

In this work the mean value of the random variable $T_{A}^{i}$ is approximated by the average of *M* observations of its values during *M* independent random walk trials. Since the outcome of any of these observations is random, they will be indexed by an element of the random path sample space. The subscript *j* = 1, *. . ., M* indexes the *j*th trial. The *M* trials are conducted so that the corresponding random variables *${\left\{T_{A}^{i}({\hat{w}}_{j}\right\}}_{j=1,...M}\left.\right\}$*are independent with the distribution identical to $T_{A}^{i}$ for every subset *A* ⊆ *V* and every node *i*. The Monte Carlo approximation of 𝔼[$T_{A}^{i}$] for a given *i* ∈ *V*, with large enough *M* and subset *A*, is expressed as

$𝔼\left[T_{A}^{i}\right]\approx \frac{1}{M}\sum_{j=1}^{M}T_{A}^{i}\left({\hat{\omega }}_{j}\right).$(2)

Since 𝔼[$T_{A}^{i}$] = 𝔼[$T_{A}^{i}\left({\hat{\omega }}_{j}\right)$], the well-known strong law of large numbers [10] states that with probability one, the sum on the right hand side of (2) converges in the limit as *M* →∞ to the expected value on the left hand side. Thus for large enough *M*, the approximation is justified. To clarify the setting for the strong law of large numbers, assume $T_{A}^{i}\left({\hat{\omega }}_{j}\right)$ is calculated for all subsets *A* and for every *i* ∈ *V*, with |*A*| ≤ *k* in parallel in a single random trial ($T_{A}^{i}$= 0 when *i* ∈ *A*). Then, ${\hat{\omega }}_{j}\in 𝕎$, where 𝕎 is defined as ${𝒫}^{2^{k}}$ and *𝒫* = Ω^∞^ is the sample space for Markov chain paths. The probability in the strong law of large numbers refers to the probability measure on 𝕎. We will also refer to an individual *${\hat{\omega }}_{j}$*as a realization.

## 3.2 Set and Graph Metrics

The distance between two vertices *a, b* ∈ *V* is denoted *d*(*a, b*). *N*[*a*] denotes the closed neighborhood of vertex *a*, given as $N\left[a\right]≐\left\{b\in V|d\left(a,b\right)\leq 1\right\}$. (Since we are focusing on unweighted graphs, adjacent nodes are a unit distance apart.) The *p*th level neighborhood (*i.e.*, the vertices at most distance *p* away) is given as $N^{P}\left[a\right]≐\left\{b\in V|d\left(a,b\right)\leq p\right\}$. These definitions are extended to arbitrary subsets *A* ⊆ *V* as $d\left(A,b\right]\;≐\min_{\mathrm{a\in A}}d\left(a,b\right)\text{ and }N^{p}[A]≐\cup_{a\in A}N^{p}\left[a\right]$.

If *S* is a set, then *S*|*_k_* is the collection of all subsets of *S* of size$k,S{|}_{k}≐\left\{A\subseteq S\left|A\right|=k\right\}$. If *f* is a function that maps domain *D* to range *R*, *f: D* → *R*, then it can be applied to any subset of *D* to generate a set of values. That is, for *S* ⊆ *D*, *f* (*S)* ≐ { *f* (*x*), *x* ∈ *S*}. For a finite set, max*_x_*_∈_*_S_ f* (*x*) returns the maximum value of *f* over domain *S*, and

$\underset{x\in S}{\arg\max}f\left(x\right)≐\left\{s\in S,f\left(s\right)=\max f\left(S\right)\right\}$ (3)

denotes the elements of *S* where the maxima of *f* occur. This notion can be extended to the top largest *k* values of *f*, 

$\underset{x\in S}{\arg\max}\left(k\right)f\left(x\right)≐\left\{T\subseteq S,\left|T\right|=k,\forall x\in T,\forall s\in S\backslash T,f\left(x\right)\geq f\left(s\right)\right\}$ (4)

which returns all possible sets of the top largest *k* values. A similar definition can be described for argmin and argmin(*k*). Note that max *f* is a single value, while argmax *f* is a set of values, and argmax(*k*) *f* is a set of sets of values. Since any *x* ∈ argmax *f* is a maximum of *f*, one can write *x* ← argmax *f* to select one of its elements. Similarly, *x* ← argmax(*k*) *f* selects a unique group of top-*k* performers.

To reduce the search for optimal sets, a set function is used as a screen, to eliminate candidate sets that are too isolated from their complements. The set function *C*: 2*^V^* → *ℛ*, called the *farness metric* measures how well any subset of *V* covers the graph by taking the sum of the distances between the set and points outside of the set. Thus given a subset *A* ⊆ *V*, 

$C\left(A\right)≐\sum_{v\in A\backslash V}d\left(A,v\right),d\left(A,v\right)=\underset{x\in A}{\mathrm{min d}}\left(x,v\right)$(5)

Smaller values of *C*(*A*) indicate more desirable coverage properties of *A*. It is not hard to show that *C*(*A*)*/*(|*V* | - |*A*|) ≥ 1 with equality when *A* is a one dominant set, *i.e.*, every element of *A^c^* has a neighbor in *A*. If *A* is a$\left\lfloor t\right\rfloor$dominant set, *i.e.*, if every vertex *v* is within ⌊*t*⌋ steps of *A*, then *C*(*A*)*/*(|*V* | - |*A*|) ≤ *t*.

## 4 Problem Setting

To motivate the optimization problem for which the solution will define an effective influencer, we will discuss the link between the time to consensus in a network containing a set *A* of leader (stubborn) agents that refuse to form consensus, and the mean first hitting time (or arrival time) to *A* of a random walk on the underlying graph.

The information state of a network at a discrete time *n*, where *n* = 0, 1, *. . .*, is represented by a vector *U_n_* ∈ ℝ*^N^* in which the *i*th component is a real number $u_{n}^{i}$ that represents the information state of node *i* at time *n*. The dynamics of the consensus process in the network for *n* ≥ 1 are given by

$u_{n}^{i}=\left\{\begin{array}{l} c\geq 0,\text{ if }i\in A\\ \sum_{j∼i,j\notin A}P\left(i,j\right)u_{n-1}^{j}+c\sum_{j∼i,j\in A}P\left(i,j\right)\;i\notin \text{A} \end{array}\right.$(6)

The initial value *U*_0_ at time *n* = 0 is,

$u_{0}^{i}\left\{\begin{array}{l} c\text{ if }i\in A\\ 0\text{ if }i\notin \text{A} \end{array}\right.$(7)

Although these values are assigned for convenience there is no loss of generality in doing so because the equations are linear.

Equation (6) states that the information value at node *i* changes to the information value at node *j* if a random walker steps from *i* to *j* in a single time step. In particular, if *j∈ A*, the value at *i* changes to *c* and is unchanged thereafter. Thus $u_{n}^{i}$ is the expected information value at *i* at time *n* after a single random walk step. From now on, assume that the nodes are ordered so that the elements of *A* appear first followed by nodes in *A^c^*.The matrix *P* can be written as:

$P=\left(\begin{array}{cc} I & O\\ R_{A} & P_{A} \end{array}\right)$(8)

The matrix *I* is the |*A*| × |*A*| identity matrix, and *O* is an |*A*| × *N* - |*A*| matrix of zeroes. The matrices *R_A_* and *P_A_* have dimensions *N* - |*A*| × |*A*| and *N* - |*A*| × *N* - |*A*|, respectively. Equation 8 then suggests that if,

$U_{n}=\left(\begin{array}{l} c1\\ x_{n} \end{array}\right)$ (9)

then *x_n_* satisfies the equation,

*x_n_*_+1_ = *P_A_x_n_* + *cR_A_***1**, *x*_0_ = 0 (10)

Here *P_A_*(*i, j*) = *P*(*i, j*) when *i, j* ∈ *A^c^*, and *R_A_*(*i, j*) = *P*(*i, j*), when *i* ∈ *A^c^* and *j* ∈ *A*. Since the transition matrix *P* (see Sec. 3) is stochastic, *R_A_* = *I* - *P_A_*. Here *I* is the *N* - |*A*| × *N* - |*A*| identity matrix. From Eq. (10), it is then clear that *x*_1_ = *cR_A_***1** = *c*(*I* - *P_A_*)**1**, and it is not hard to establish by mathematical induction that

*x_n_* = *c*(*I* -$P_{A}^{n}$)**1** (11)

so that the substochastic property of *P_A_* implies that *x_n_* → x* as *n* →∞, with x* = *c***1**. In this sense, the information value common to nodes at *A* spreads to the rest of the network.

We can define the rate of convergence to consensus to be the rate of convergence of the *l*_1_ difference ||x* - *x_n_*||_1_ to 0. The following equation shows that the rate is controlled by the tail of the distribution of the first hitting time to *A*.

||x* - *x_n_*||_1_ = ℙ[*T_A_ > n*] (12)

The proof of Eq. (12) can be found in the Appendix. It uses standard results in the theory of absorbing Markov chains. For finite irreducible Markov chains, it is known that the right-hand side decays exponentially in *n*. For an arbitrary initial distribution *µ* on states of the chain one can show ([15] Chapter 3 and [13] Chapter 2) that

$P_{µ}\left[T_{A}>n\right]\leq \exp\left(-\left\lfloor \frac{n}{\mathrm{\lceil e}t_{A}^{*}\rceil }\right\rfloor \right)$(13)

where $t_{A}^{*}$ = max*_i_*_∈_*_V_* 𝔼*_i_*[*T_A_*]. Now note that,

$\underset{i\in V}{\max}{𝔼}_{i}\left[T_{A}\right]\leq F\left(A\right)=\sum_{i\in V}E_{i}\left[T_{A}\right]\leq \left(N-\left|A\right|\right)\underset{i\in V}{\max}E_{i}\left[T_{A}\right]$(14)

For fixed cardinality |*A*|, the inequality in Eq. (14) shows that

$F\left(A\right)=\sum_{i\in V}{𝔼}_{i}\left[T_{A}\right]$ (15)

lies within the same bounds as the actual rate of convergence to consensus. Thus our strategy for finding the set that maximizes the rate of convergence to consensus and therefore finding an effective influencer is to seek the set *A* that is a solution to the following optimization problem:

$\underset{\begin{array}{l} A\subseteq V\\ \left|A\right|=k \end{array}}{\min F\left(A\right)}$ (16)

In earlier work [16], a method of solving or approximating the solution of Eq. (16) used the fact that *F*(*A*) can be computed by inverting the matrix *P_A_*. Although the method is effective for graphs of smaller size up to hundreds of nodes, the number of inversions required make it impractical to use for the graphs we consider in this paper.

## 5 Multi-stage Approximation Algorithms

The outline of two scalable procedures for solving Eq. (16) is presented below. Since *F*(*A*) is a discrete function, an exact solution to this minimization problem would require $\left(\begin{array}{l} \left|V\right|\\ k \end{array}\right)$ evaluations. For realistic problem sizes, this is computationally infeasible. For example, to find the best *k* = 100 influencers in a million node network would require the examination of $\left(\begin{array}{l} 1,000,000\\ 100 \end{array}\right)$ ≈ 10^442^ different subsets as possible solutions. For this reason we present multi-stage heuristics to solve this problem. They reduce the search space of possible solutions by focusing on candidate sets constructed with vertices of large degree (hubs). The idea here is that the hubs can act as attractors for hitting sites of random walks.

A Monte Carlo approximation of *F* is calculated for each such set and minimization over the starter class produces an offered approximation to the exact solution. Thus, given a network graph *G* = (*V, E*) and an integer *k*, we approximate the *k*-element optimal influencer set (target set) with the smallest aggregate arrival time as follows:

1. From an initial set of *h* top hubs, *b* target sets of cardinality *k* with the largest aggregate *p*-neighborhood size are selected.
2. The farness value *C*(*A*) of each target set *A* is computed and is used to refine the class of sets produced in the previous step. Thus the function *C* is used as a conservative screen to reduce the number of feasible sets that must be examined for computing *F*(*A*).
3. The sets are ranked by their respective *C* values with the top *Q* target sets having the smallest value of *C*.
4. A finite sample approximation of *F* based on *M* random walk simulations, *F_M_*, is obtained for these sets. A preliminary ranking of these sets is based on choosing sets with the smallest calculated value.
5. A new ranking based on a finite sample approximation of *F_M_* for larger *M* is produced from the best sets in the previous step
6. The set *$\hat{A}$* with the smallest value *F_M_*(*$\hat{A}$*) is offered as the approximate solution of the minimization problem in Eq. (16).

**Remark:** Since for any set *A*, *C*(*A*) ≤ *F*(*A*), *C* is called a conservative screen.

The number of hubs, *h*, to consider is the first step in limiting the search space. In practice, *h* is a small multiple of *k* and hence much smaller than |*V* |. Thus, we reduce a $\left(\begin{array}{l} \left|V\right|\\ k \end{array}\right)$search into one of size $\left(\begin{array}{l} h\\ k \end{array}\right)$ . The evaluation of neighborhoods can be either first generation (*i.e.*, friends) when *p* = 1 or second generation (*i.e.*, friends and friends of friends) when *p* = 2 or higher. Typically we use a *p* = 1 level neighborhood *N*[*A*] for large networks, although for small networks (|*V* | *<* 100) a *p* = 2 level neighborhood *N*^2^[*A*] calculation can sometimes yield improved results.

Furthermore, two variations of this algorithm are considered, one for small values of *k* for which it is practical to evaluate all *^$\left(\begin{array}{l} h\\ k \end{array}\right)$^ k*-element subsets, and a second semi-greedy algorithm that computes a solution incrementally. The first version of the algorithm, for small *k*, requires six parameters

⚫ *k* the number of elements in target sets to find;
⚫ *h* the number of top hubs to analyze as candidate vertices;
⚫ *p* the *p*th-level neighborhood to consider for maximal aggregate coverage;
⚫ *b* the number of top *k*-element subsets with which to compute farness metric;
⚫ *q* the number of top farness-metric subsets over which to compute random walks; and
⚫ *M* the number of trials to use when computing the Monte Carlo random walks

The parameter *h* controls the depth of the search and serves as a rough measure of quality versus time trade-off for the heuristic. With smaller values of *h*, the algorithm is faster, while maximizing *h* to be the number of vertices in the graph ensures we perform a complete exhaustive search on all $\left(\begin{array}{l} \left|V\right|\\ k \end{array}\right)$ possible target sets. In practice, *h* is often a small multiple of *k*, *p* = 1, *q* ≤ *b*, and an approximation for *M* can be determined with the analysis in the next section. In the networks presented in Sec. 7, for example, *h* is roughly 8 to 10 times the value of *k*, and *b* = *q* = 5.

**Fig. 1:**
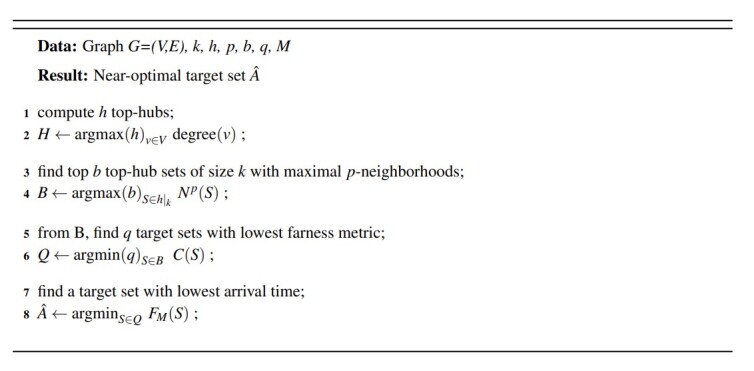
Combinatorial algorithm for evaluation of top-hubs.

**Fig. 2:**
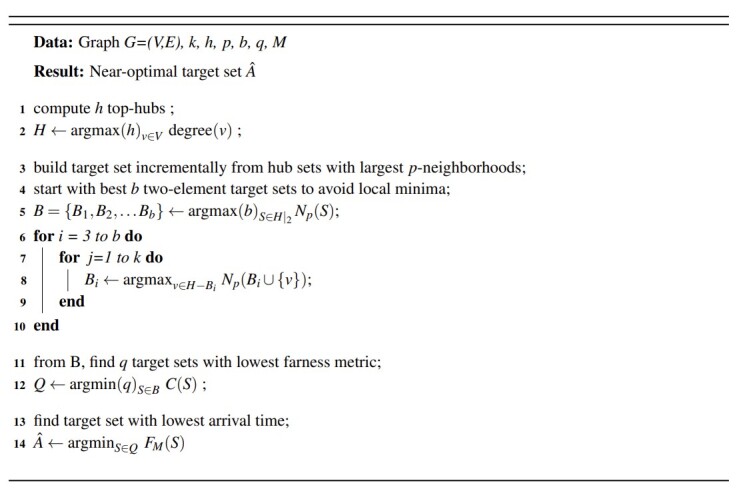
Semi-greedy algorithm for evaluation of top-hubs.

For large values of *k*, the second version of the algorithm uses a greedy method to construct *A*. Rather than examine all $\left(\begin{array}{l} h\\ k \end{array}\right)$ possible subsets, we begin with a construction set *A*´ containing the two-element set with largest *N^p^*[*A*´] and continually add hubs from the candidate list that offer the largest neighborhood growth to *A*´. (The motivation for starting with a two-element set is to avoid local minima, which are potentially created by always starting with the largest hub.) The algorithm terminates with *$\hat{A}$*= *A*´ when|*A*´| = *k*, and reports the farness metric *C*($\hat{A}$), together with approximation *F_M_*($\hat{A}$). The algorithms are formally described in Figs. 1 and 2, using notations described in Eq. 3 and Eq. 4 in Sec. 3.2.

To summarize, the algorithms differ only in the first two sections (lines 1-4 in Fig. 1, and lines 1-10 in Fig. 2). The combinatorial algorithm examines $\left(\begin{array}{l} h\\ k \end{array}\right)$ candidate sets, while the semi-greedy version examines roughly $\left(\begin{array}{l} h\\ k \end{array}\right)$+ *hk* candidate sets.

**Data:** Graph *G=(V,E), k, h, p, b, q, M*
**Result:** Near-optimal target set $\hat{A}$
1 compute *h* top-hubs;
2 *H* ← argmax${\left(h\right)}_{v\in V}$degree(*v*) ;
3 find top *b* top-hub sets of size *k* with maximal *p*-neighborhoods;
4 *B* ← argmax${\left(b\right)}_{S\in h{|}_{k}}$ *N^p^*(*S*) ;
5 from B, find *q* target sets with lowest farness metric;
6 *Q* ← argmin${\left(q\right)}_{S\in B}$C(S);
7 find a target set with lowest arrival time;
8 $\hat{A}$ ← argmin*${\left(\mathrm{argmin}\right)}_{S\in Q}$F_M_*(*S*) ;

## 6 Stochastic Analysis

To understand what a finite sample approximation of *F* is, recall from Sec. 3 that for any subset *A* we have a finite sample approximation of **E**[$T_{A}^{i}$] for *i* ∈ *A^c^*, from Eq. (2) Therefore we can define a finite sample approximation for *F* as:

${ℱ}_{M}\left(\omega ,A\right):=\sum_{i\in A^{c}}\frac{1}{M}\sum_{j=1}^{M}T_{A}^{i}\left({\hat{\omega }}_{j}\right)$(17)

where *ω *$=\left({\hat{\omega }}_{1},...,{\hat{\omega }}_{j},...{\hat{\omega }}_{M}\right)$ ∈ 𝕎*^M^*. Note that Eq. (17) implies that 𝔼[*ℱ_M_*(*ω, A*)] = *F*(*A*). For a fixed *ω* (*i.e.*, a realization of the random variable), *ℱ_M_*(*ω, A*) is a non-random real number for each set *A*. We can therefore pose the finite sample analogue of the optimization problem Eq. (16) as

$\underset{\begin{array}{l} A\subseteq V\\ \left|A\right|=k \end{array}}{\min}{ℱ}_{M}\left(\mathrm{\omega ,}A\right)$ (18)

The problem in Eq. (18) is a Monte Carlo approximation of the original discrete optimization problem and its solution is a sample average approximation of the solution of (16). The algorithms outlined in Fig. 1 and Fig. 2 produce approximate solutions of Eq. (18).

To determine the effectiveness of the algorithms, we address two questions

1. Given any set *A*, how accurately does *ℱ_M_*(*ω, A*) approximate *F*(*A*)?
2. Let *$\hat{A}$* be an offered solution of Eq. (16) obtained by the algorithm. How can its quality be assessed?

There is a body of research devoted to a discussion of methods of solution and relevant convergence theory for this class of problem for both continuous and discrete optimization problems. References can be found in the survey by Homem-de Mello and Bayraksan [17]. Kleywegt *et al.* [18] discussed the convergence of the approximation in the context of discrete optimization problems. Recently, Lee, Hasenbein and

**Fig. 2.** Semi-greedy algorithm for evaluation of top-hubs.

Morton [19] applied the methods of [17] and of Mak *et al.* [20] to a class of discrete problems arising from the optimal detection of a virus spreading in a contact network.

For us, the first question can be handled by standard statistical methods. Indeed, since our estimate of *F* can be written as a sum of identically distributed random variables, as in Eq. (17), a statistical comparison with the sample mean of *ℱ_M_* and *F* can be obtained using normal distribution theory.

In the next section we quote the result of Klewegt *et al.* [18] to show the convergence of good approximations to the solution of the problem in Eq. (18) to good approximations of the problem in Eq. (16) as *M* →∞, a consequence of the strong law of large numbers. In our situation a "good" approximation of the problem Eq. (18) is a set *A* for which finite the sample approximation value *ℱ_ℳ_* (*ω, 𝒜*) is close to the minimum value of *ℱ* (*ω,* ·) in some pre-specified sense. We can judge a set *A* to be a good solution of the problem in Eq. (16) in an analogous way. In the stochastic optimization literature the mathematical definition of good is based on the concept of *ε*-optimality. We will briefly mention this concept later in our discussion. Consequently, if it can be demonstrated that our offered solution is an *ε*-optimal solution of the problem in Eq. (18), then using Kleywegt's result, we can establish that it is an *ε*-optimal solution of Eq. (16), the original problem.

A standard method for assessing the quality of the approximation, and therefore answering question 2, is to estimate the so-called optimality gap [21]. Unfortunately, this method requires highly accurate knowledge of the minimum value in Eq. (18) or reasonable confidence intervals for that value. For large realistic graphs the amount of calculation needed to obtain this information makes this approach impossible. Even boot-strapping or other sub-sampling techniques that depend on the ability to evaluate or approximate adequate samples of *F* are ruled out.

Rather than centering our efforts on obtaining a provably good approximate solution of the problem in Eq. (16), we observe that the repeated ranking of sets in the algorithms can be interpreted as methods of calculating elements in the lower tail of the distribution of values of *F*(*A*) over all subsets *A* of fixed cardinality. Given a small positive *η* suppose *c* is defined by the relation *Prob*[*F*(*A*) ≤ *c*] = *η*. Here "*Prob*" refers to the probability distribution generated by the histogram of values of *F*(*A*) for |*A*| = *K*. The minimum value of *F* is of course located in the lower tail. We will arrange the value of *c* so that the offered solution and the actual optimal value are both in the interval [min_|_*_A_*_|=_*_k_ F*(*A*), *c*] (see the Appendix). If a subset *A* is just selected at random it will have a larger value of *F* and thus be a worse performer with probability 1 - *η*. In other words with high probability, our offered solution of Eq. (16) has an upper bound of *c* and ranks in the 100 × (1 - *η*)th percentile of sets as ranked by effectiveness of spread. This gives some precision to the conclusion of the heuristic calculations described in the next section.

Our proposed first step toward answering question 2 then is to provide an estimate and confidence interval for *c*. We cannot guarantee that *c* is arbitrarily close to the minimum of *F*, but given *η*, we can reliably estimate the corresponding *c* in a statistical sense. The examples in Sec. 6 show that the semi-greedy algorithm produces a set of high quality target sets (*K* = 10), from large graphs in a matter of milliseconds. It is therefore feasible to generate many independent samples from the left tail of the distribution of {*F*(*A*)}_|_*_A_*_|=_*_K_*. A value of *η* can be selected. If an estimate of *c* is calculated from these samples, Chebyshev's inequality can be used to estimate the probability that the estimate is accurate. A full development of this idea will be the subject of future work, but some details can be found in the Appendix. Variants of this approach are standard in the design of randomized algorithms [22], but see also Ref. [11] for application to bootstrap-type methods.

## 6.1 Convergence Results

Setting *$H\left({\hat{\omega }}_{j},A\right)=\sum_{i\in A^{c}}T_{A}^{i}\left({\hat{\omega }}_{j}\right)$*, we rewrite Eq. (17) as

${ℱ}_{M}\left(\omega ,A\right)=\frac{1}{M}\sum_{j=1}^{M}H\left({\hat{\omega }}_{j},A\right)$(19)

then one can see that *ℱ_M_*(*ω, A*) is a sum of *M* independent and identically distributed (i.i.d.) random variables, {*H*(*${\hat{w}}_{j}$, A*)} *_j_*_=1_*_...M_*, with **E**[*H*(${\hat{w}}_{j}$, A)] = *F*(*A*). Therefore, the sum will converge to *F*(*A*) with probability one as *M* →∞ by the strong law of large numbers. Moreover [18], if we set

$v^{*}=\underset{\left|A\right|\leq k}{\min}F\left(A\right)$

$v_{M}\left(\omega \right)=\underset{\left|A\right|\leq k}{\min}{ℱ}_{M}\left(\omega ,A\right)$ (20)

then *v_M_*(*ω*) → v* with probability 1 as *M* →∞, as a consequence of the finiteness of the class of feasible sets ([18], Proposition 2.1). The *ε*-optimal sets for Eq. (16) and Eq. (18) are very closely related. In particular, if *ε >* 0 is given, a set *A* is *ε*-optimal for Eq. 16) if *F*(*A*) ≤ v* + *ε*. There is an analogous definition for the *ε*-optimal set for Eq. (18). Kleywegt *et al.* showed that for fixed *ε* and large enough *M*, if *A* is an *ε*-optimal solution for Eq. (18), then it is an *ε*-optimal solution of Eq. (16).

A statistical estimate of the accuracy of the deviation of *ℱ_M_* from *F* is based on a routine application of the central limit theorem. Equation (17) shows that for each *i* ∈ *V* and *A* ⊆ *V*, the random variable *H*(*ω, A*) in Eq. (19) has a distribution with exponential tails since it is a sum of random variables *$T_{A}^{i}$* with exponential tails (see Sec. (4) and note that $T_{A}^{i}$ = 0 when *i* ∈ *V*). Thus, all moments of the distribution for *H* exist and the central limit theorem applies. Asymptotically in the limit of large *M*, we have the following Monte Carlo estimate as the answer to question 1:

$\mathbb{P}\left\{{ℱ}_{M}\left(\omega ,A\right)-\frac{st_{\alpha }}{\sqrt{M}}\leq F\left(A\right)\leq {ℱ}_{M}\left(\omega ,A\right)+\frac{st_{\alpha }}{\sqrt{M}}\right\}\approx 1-\alpha$(21)

The variable *s* is the sample standard deviation

$s^{2}=\frac{\left({\sum_{j=1}^{M}\left[H\left({\hat{\omega }}_{j},A\right)-\bar{H}\right]}^{2}\right)}{M-1}$(22)

where *$\bar{H}$*(= *ℱ_M_*(*ω, A*)) is the sample mean of *H*, and where *t_α_* is the value from the *t*-distribution with *M* -1 degrees of freedom required for a confidence interval at level *α*. The maximum deviation *δ* from *F*(*A*) defined in Eq. (21) requires a sample size *M* = ($\frac{st_{\alpha }}{\delta }$)^2^.

## 7 Examples and Results

In this section we describe how these optimization algorithms for approximating min_|_*_A_*_|=_*_k_ F*(*A*) are used in practice. We consider examples based on real networks taken from the application areas of biology, sociology, chemistry, computer science, and information technology. They include examples of animal interactions, human connections, co-authorships of technical publications, collaboration in music, protein-protein interactions, and autonomous systems. Table 1 list the networks used in this study. Unless otherwise noted, we consider their topology as undirected, connected graphs by treating edges as bidirectional, utilizing only the largest connected component, and removing any multi-edges or self-loops. Examples were collected from network databases Konect [23], SNAP [24], and UC Irvine [25]. In addition, several webgraph topologies were generated from examining public web sites.

Finding the best *k*-element target set *A* that minimizes *F*(*A*) is computationally challenging, and examining all $\left(\begin{array}{l} V\\ k \end{array}\right)$ possible subsets is only feasible for small graphs. In evaluating the quality of the approximations, we take a two-pronged approach:

1. Compare the approximation to the true optimal by exhaustive search for tiny graphs (*V <* 50) and small values of *k* (Sec. 7.2).
2. For larger *V* and *k*, compare the approximate solution to the expected value of *F*(·) by direct computation (whenever feasible) or by sampling and use of the central limit theorem (Sec. 7.3).

In these network examples, the approximation algorithms (Sec. 5) were implemented with the following parameters: *p* = 1, *h* = 10*K,* and *b* = *q* = 5. This offered a practical compromise between quality of solution and reasonable computation time.

## 7.1 Case Study: *Caenorhabditis elegans* Neural Network

To illustrate these ideas, consider the neural network of the *Caenorhabditis elegans (C. elegans)* microscopic roundworm [23]. For this experiment, the topology of its nervous system is represented as an undirected, unweighted connected graph of 297 vertices and 2,148 edges. It appears about half-way down the list of example networks listed in Table 1 and is large enough to be interesting but small enough that we can find optimal target sets by exhaustive search, for small values of *k*. For *k* = 3, there are $\left(\begin{array}{l} 297\\ 3 \end{array}\right)$ or 4,322,340 possible three-element subsets to evaluate *F*(*A*). For clarity, we denote *F_k_*() to the set of all function values for |*A*| = *k*, A* to be the true optimal, and $\hat{A}$ to be the solution offered by the heuristic.

**Table 1:** Application network topologies used in this study (largest connected component of undirected versions). The average degree is (*d*) and the maximum degree is Δ.

Network	Application	|*V* |	|*E*|	(*d*)	Δ	Δ*/*(*d*)
Zebra	animal contact network	23	105	9.13	14	1.5
Dolphin	animal contact network	62	159	5.13	12	2.3
Terrorist	network social network	64	243	7.59	29	3.8
High School	social network	70	274	7.83	19	2.4
MIT students	mobile social network	96	2,539	52.90	92	1.74
Hypertext 2009	social interaction	113	2,196	38.9	98	2.5
Florida ecosystem wet	food network	128	2,075	32.42	110	3.4
PDZBase	metabolic network	161	209	2.59	21	8.1
Jazz	collaboration network	198	2,742	27.79	100	3.6
GE 200	top-level web graph	200	1,202	12,02	124	10.3
Chevron 200	top-level web graph	200	5.450	54.50	189	3.5
Abilene218	computer network	218	226	2.07	10	4.8
Bethesda	top-level web graph	255	422	3.31	81	24.5
C. Elegans	neural network	297	2,148	14.46	134	9.3
NetScience	co-authorship	379	914	4.82	34	7.0
Arenas-email	communications network	1,133	5,451	9.62	71	7.4
FAAa air traffic	infrastructure	1,226	2,408	3.9	34	8. 6
Human protein	protein interaction	2,217	6,418	8.94	314	26.5
ca-GrQc	co-authorship	4,158	13,422	6.46	81	12.5
ca-HepTh	co-authorship	8,638	24,806	5.74	65	11.3
ca-HepPh	co-authorship	11, 204	117,619	23.38	491	21.0

a FAA = Federal Aviation Administration.

**Fig. 3:**
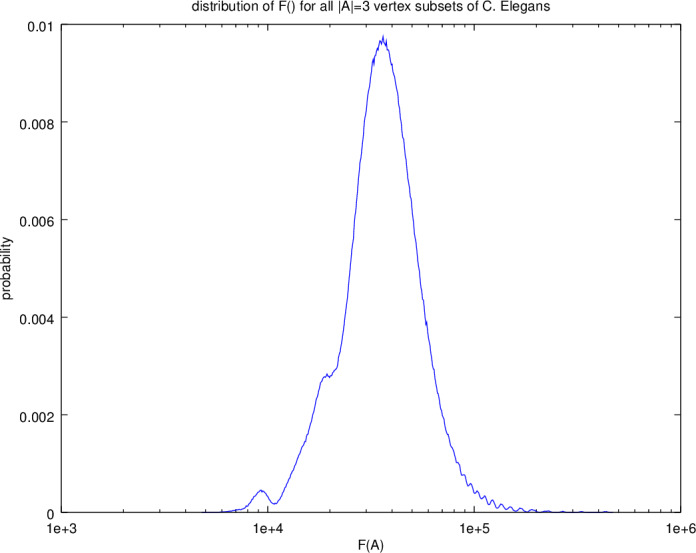
Distribution of *F*(·) for all three-element vertex subsets of *C. elegans*. Optimal value *F*(A*) is 4.85 × 10^3^, mean is 38.9 × 10^3^, and a maximum value is 470 × 10^3^ The semi-greedy algorithm produces a value *F*($\hat{A}$) of 5.09 × 10^3^, within 5% of optimal, or ranking in the top 0.00007%.

Figure 3 illustrates the distribution of these values on a logarithmic *x*-axis. Most values of *F*_3_() fall between 30 × 10^3^ and 90 × 10^3^, with a mean (expected value) 𝔼[*F*_3_()] of 38.9 × 10^3^. Table 2 lists the top 10 target sets, together with their respective *F*(*A*) values. The near-optimal approximation $\hat{A}$ ranks third among 4,322,340 possible target sets, representing the top 0.0007%. and within 5% of the true optimal solution, A*. The remaining target sets in the top 10 list are also excellent candidates, representing an almost eight-fold improvement over the expected value (𝔼[*F*_3_(·)]*/F*(A*) = 7.6).

## 7.2 Comparison to True Optimal (Small Networks)

For tiny graphs and modest target sets (for example, |*V* | between 10 and 100 and *k <* 10) it is possible to compute the optimal target set by exhaustive search. **Table 3** shows the relative error of the estimates from the true optimal, in the form of |*F*(A*) - *F*($\hat{A}$)|*/F*(A*), for *k* values of 1 through 10. The cases where the heuristic is exact (*F*(A*) = *F*($\hat{A}$)) are represented by an asterisk.

In such cases, the heuristics find the true optimal about a third of the time (shown in Table 3 as asterisks) while they often produce values with 10% to 20% relative error from the true optimal. Thus, the heuristics are a mixed bag for tiny graphs, although they do produce estimates that are significantly smaller than their expected value (Sec. 7.3). The heuristics perform reasonably well, but they are not optimized for these

graphs because the hubs are very small compared to the average degree and hence are not as distinguishable.

In rare instances, the heuristic methods outlined in Sec 5 perform worse than building an optimal target set from the top *k* hubs. For example, in the **MIT students** network, the top *k* hubs perform about 15% better for target sizes from 1 through 10. Such cases are uncommon, but adding a test for this is computationally trivial. Thus, the updated heuristic meethod provides a target set that produces the minimum of the two.

## 7.3 Comparison to Expected Value (Larger Networks)

The semi-greedy algorithm presented in Fig. 2 is more appropriate for medium and larger-sized graphs and significant *k* and *h* values, as discussed at the end of Sec. 5. For these network sizes, the hubs are more distinguishable and the heavy-tailed nature of the graph's degree distribution plays an important part in the optimization strategy. The true optimal is not available for these larger networks, so we must find other characteristics to compare our heuristics against. One important attribute is the expected value of *F*(·), which gives an indication of what an average random guess might produce.

Given a fixed *k*, the expected value of *F*(·), 𝔼[*F_k_*()], is the mean value of *F*(·) for all |*A*| = *k* subsets of *V* . That is,

$𝔼\left[F_{k}()\right]=\frac{1}{\left(\begin{array}{l} V\\ k \end{array}\right)}\sum_{\left|A\right|=k}F\left(A\right)$(23) 

**Table 2.** Top three-element target sets, with respective *F* (·) values. The near-optimal approximation A* is within 5% error of true optimal solution, A* .

A			F(A)		
A*→	{3,	14,	182}	4:85×10^3^	←F(A*)
	{5,	14,	182}	5.03×10^3^	
$\hat{A}\to$	{14,	44,	182}	5.09×10^3^	$\leftarrow F\left(\hat{A}\right)$
	{14,	18,	182}	5.11×10^3^	
	{4,	14,	182}	5.11×10^3^	
	{3,	4,	182}	5.16×10^3^	
	{3,	5,	182}	5.17×10^3^	
	{3,	18,	182}	5.18×10^3^	
	{14,	52,	182}	5.20×10^3^	
	{14,	75,	182}	5.28×10^3^	

**Table 3:** Quality of near-optimal *F*( $\hat{A}$) estimates compared to the true optimal, *F*(A*), as a relative precent error. Asterisk * denotes that exact optimal is achieved: 0.0% error.

Network	k=1	2	3	4	5	6	7	8	9	10
zebra	*	8.1	*	*	4.4	4.7	2.2	7.5	21.3	36.0
dolphin	*	*	0.5	1.6	1.6	5.9	5.1	4.3	4.8	6.1
terrorist	*	*	20.9	26.9	13.1	11.8	9.7	7.6	11.7	7.2
high school	*	*	*	*	*	*	2.7	1.0	0.5	5.2

is a convergent sum, since the domain of *F_k_*() is finite. If we have all values of *F_k_*() then the process is a simple averaging. For large networks, however, we can approximate the expected value via sampling and an application of the central limit theorem. With a sample size of *L >* 30 the approximate expected value of *F_k_*() is the average of randomly sampled values of *F_k_*(*A*) (with replacement), and its standard deviation is scaled by 1*/ $\sqrt{L}$*. That is, if the samples means have an average of *µ* and a standard deviation of *σ*, then the distribution of the sample means will form a normal distribution, with a mean of *µ* and a standard deviation of *σ/$\sqrt{L}$*. Letting *A_r_*_(_*_i_*_)_ denote a randomly indexed vertex set *A* from all possible subsets $\left(\begin{array}{l} V\\ k \end{array}\right)$, and 𝔼*_L_*[*F_k_*()] denote the approximate expected value from a large sample size *L*, we can write

$𝔼\left[F_{k}()\right]\approx {𝔼}_{L}\left[F_{k}()\right]=µ=\frac{1}{L}\sum_{i=1}^{L}F_{k}\left(A_{r(i)}\right)$ (24)

In our experiments we use *L* = 1, 000 to ensure a reasonably tight bound on the approximation 𝔼[*F_k_*()] ≈ *µ* with standard deviation of *σ/ $\sqrt{L}$*.

For example, in the *C. elegans* network, a random sampling of 1,000 *F*(*A*) values yields an approximate 𝔼[*F_k_*()] value of 38.8 × 10^3^ with a standard deviation of 0.63 × 10^3^ or ±1.6%, compared to the true 𝔼[*F_k_*()] value of 38.9 × 10^3^. In this case, the quality of the heuristic over the expected value, 𝔼[*F_k_*()]*/F*($\hat{A}$) ≈ (38.8 × 10^3^)*/*(5.09 × 10^3^) or about 7.6 times smaller.

**Table 4:** Improvement of optimal over expected value: 𝔼[*F_k_* ()]*/F*(A*)

Network	k=1	2	3	4	5	6	7	8	9	10
zebra	3.5	2.3	2.2	2.0	1.9	1.7	1.6	1.6	1.6	1.6
dolphin	3.6	3.6	3.4	3.2	3.0	3.0	2.8	2.8	2.8	2.8
terrorist	9.2	5.4	5.0	4.2	4.0	3.7	3.6	3.5	3.5	3.5
high school	3.1	2.5	2.5	2.4	2.3	2.3	2.2	2.1	2.0	2.0

**Table 4** presents this ratio for the smaller networks, where we compute 𝔼[*F_k_*()] directly. In such cases we see that the ratio of the true optimal *F_k_*(A*) to the expected value is rather small (*<* 10) throughout the various networks and *k* values. This is largely due to the topology of the networks and their diminutive size.

Nevertheless, as networks increase in size, this ratio can become significant. **Table 5** shows that the *F*($\hat{A}$) estimates generated by the semi-greedy algorithm outperform the approximate expected values, sometimes by **two or three orders of magnitude** for the larger networks, so finding good estimates to the optimal solution can have significant benefits.

**Table 5:** Improvement of near-optimal over expected values for larger networks: 𝔼[*F_k_* ()]*/F*($\hat{A}$)

Network	k=1	2	3	4	5	6	7	8	9	10
MIT students	2.5	2.0	1.8	1.7	1.7	1.7	1.7	1.7	1.6	1.6
Hypertext 2009	5.2	3.8	3.2	3.1	3.1	2.9	2.7	2.7	2.7	2.5
Florida ecosystem wet	4.6	3.0	2.4	2.5	2.4	2.2	2.0	2.0	1.9	1.8
Chevron 200	8.1	4.6	3.4	2.7	2.5	2.3	2.0	1.7	1.6	1.6
GE 200	19	13	10	7.3	5.9	5.0	7.8	6.6	6.3	7.3
Abilene218	2.8	2.3	2.4	2.3	2.2	2.1	2.1	2.0	1.9	2.0
Bethesda	54	30	22	19	16	14	13	12	12	11
Jazz	9.9	5.0	3.8	3.1	2.9	2.8	2.6	2.4	2.4	2.2
NetScience	4.6	4.6	6.6	6.4	5.5	5.8	5.1	5.2	4.9	4.7
C. Elegans	27.4	9.6	7.6	5.9	5.9	5.1	5.1	5.0	4.4	4.4
Arenas-email	19.8	12.1	8.8	6.4	7.1	6.6	5.8	5.9	5.8	5.4
FAAa air traffic	16	11	9.2	7.9	6.7	6.1	5.6	5.4	6.0	6.1
Human protein	165	99	78	51	59	59	53	45	46	40
ca-GrQc	37	20	16	12	12	11	9.3	8.6	7.9	7.2
ca-HepTh	34	23	19	17	15	14	13	12	11	11
ca-HepPh	161	109	76	54	46	39	34	23	28	25

One notable pattern for real networks is that the 𝔼[*F_k_*]*/F*($\hat{A}$) ratio typically **decreases monotonically** as *k* increases. This is understandable, since the individual role of a specific vertex in that set diminishes as the target set size increases.

Another helpful observation is that the ratio of a network's maximum degree over its average degree Δ*/*(*d*) (listed in Tbl. 1) is a **good indicator** of how well the 𝔼[*F_k_*()]*/F*($\hat{A}$) ratio holds up over the *k* range. Indeed, **Table 6** reveals the relationship between these two measurements, ordered by largest 𝔼[*F*_1_()]*/F*($\hat{A}$) value and the corresponding Δ*/*(*d*) ratios, revealing a **correlation coefficient of 0.85** between these two quantities.

**Table 6:** Ordering of 𝔼[*F*_1_()]*/F*($\hat{A}$) to Δ*/*(*d*) for various networks appears to be related to how well the near-optimal solutions outperform respective expected values.

Network	$\frac{𝔼[F1()]}{F\left(\hat{A}\right)}$	Δ*/*(*d*)
Human protein	165	26.5
ca-HepPh	161	21.0
Bethesda	54	24.5
ca-GrQc	37	12.5
ca-HepTh	34	11.3
*C*. elegans	27	9.3
Arenas email	20	7.4
GE200	19	10.3
FAA air traffic	16	8.6
Jazz	9.9	3.6
Chevron2008	8.1	3.5
Hypertext 2009	5.2	2.5
NetScience	4.6	7.0
Florida ecosystem wet	4.6	3.4
Abilene	2.8	4.8
MIT students	2.5	1.7

## 7.4 Comparison to Best Sampled Values (Larger Networks)

In sampling the *L* subsets for computing the expected value in the previous section, we can also use these *L* samples and choose the best ones (highest percentage ranking) to get an indication how well the heuristics perform. That is, with finite samples {*A_r_*_(1)_, *A_r_*_(2)_, *. . . A_r_*_(_*_L_*_)_}, we can define the smallest value encountered

𝕄*_L_*[*F_k_.*()] ≐min{*F_k_*(*A_r_*_(1)_), *F_k_*(*A_r_*_(2)_), *. . . F_k_*(*A_r_*_(_*_L_*_)_} (25)

This provides a tigher lower bound, closer to the true optimal. Note that, by definition, *F*(A*) ≤ *F*($\hat{A}$) and 𝕄*_L_*[*F_k_*()] ≤ 𝔼*_L_*[*F_k_*()]. The goal is that *F*($\hat{A}$) should be less than 𝕄*_L_*[*F_k_*()], and this occured over 98% of the time in our experiments, as described below.

**Table 7** lists this potential improvement of the offered solution *F_k_*($\hat{A}$) over sampled values. In this experiment, we use *L* = 1000 to provide an estimate of the top 0.1% ranking from these *L* samples. In nearly all instances of networks and *k* values, the ratio 𝕄*_L_*[*F_k_*()]*/F_k_*($\hat{A}$) is greater than 1.0, demonstrating that the *F_k_*($\hat{A}$) solution is not only significantly better than the expected value of 𝔼*_L_*[*F_k_*()], but it is also typically superior than the best of 1,000 random samples. For the *k* = 1 and *k* = 2 cases, *F_k_*($\hat{A}$) performs nearly as well, but for larger *k* values, the performance typically increases to a factor between **1 and 5 times better**. In only three cases out of 160 (16 networks, with 10 *k* values each) does this ratio drop below 1.0. This occurs for the *k* = 1 or *k* = 2 examples in the **MIT students**, **Florida ecosystem wet**, and **Netscience** networks, which are in the low Δ*/*(*d*) ratios, as shown in **Table 6**, and which show the most limited effect for potential increase in speed from the original optimization problem. In short, the heuristics **work best where it matters**: cases with larger *k* values, where the problem becomes harder, and networks with high Δ*/*(*d*) ratios, where the potential for finding good target sets yields the most benefit (*i.e.*, produces higher 𝔼*_L_*[*F_k_*()]*/F_k_* ($\hat{A}$) ratios).

**Table 7:** Improvement over top 0.1% ranking from 1,000 samples: *M*_1_*_,_*_000_[*F*_3_()]*/F*_3_($\hat{A}$)

network	k=1	2	3	4	5	6	7	8	9	10
MIT students	**0.9**	1.0	1.0	1.1	1.1	1.1	1.2	1.1	1.1	1.2
Hypertext 2009	1.3	1.6	1.6	1.6	1.5	1.7	1.7	1.7	1.8	1.5
Florida ecosystem wet	1.0	**0.9**	1.1	1.1	1.2	1.2	1.0	1.2	1.2	1.1
Chevron 200	1.0	1.5	1.5	1.4	1.3	1.3	1.2	1.1	1.1	1.0
GE 200	1.0	1.6	1.2	1.2	1.1	1.2	1.5	2.0	1.5	2.0
Abilene218	1.0	1.0	1.2	1.3	1.2	1.1	1.3	1.3	1.2	1.3
Bethesda	1.0	1.2	1.2	1.3	1.8	1.7	1.6	1.9	2.0	1.7
Jazz	1.0	1.2	1.2	1.3	1.4	1.4	1.4	1.4	1.3	1.3
NetScience	1.0	**0.8**	1.8	1.8	1.7	1.9	2.1	1.9	2.2	2.0
C. Elegans	1.0	1.5	1.5	1.8	1.4	1.4	1.6	1.6	1.8	1.6
Arenas-email	1.4	1.5	1.6	2.0	1.8	2.1	2.1	1.9	2.2	2.0
FAAa air traffic	1.0	1.6	2.4	2.3	2.1	2.0	1.8	2.0	2.4	2.3
Human protein	1.0	1.8	2.3	3.1	3.6	4.0	4.1	4.6	4.7	5.4
ca-GrQc	1.2	1.6	1.7	1.8	2.0	2.3	2.3	2.4	2.1	2.1
ca-HepTh	1.2	2.0	2.5	2.5	2.9	3.7	3.4	3.3	3.5	3.7
ca-HepPh	1.1	1.7	2.6	2.3	2.3	3.2	2.8	3.2	3.8	3.4

## 7.5 Timing Analysis

**Table 8** lists the computation time required to find the larget target size in our test cases (*k* = 10) for the various networks. All experiments were conducted on a common desktop workstation, running Ubuntu Linux 4.15, with an Intel^Ⓒ^ i7-3770 processor running at 3.4 GHz, and outfitted with 32 MB of RAM. ^1^1Certain commercial products or company names are identified here to describe our study adequately. Such identification does not imply recommendation or endorsement by the National Institute of Standards and Technology, nor does it imply that the products or names identified are necessarily the best available for the purpose.

The algorithms were coded in C++, and compiled under GNU g++ 7.4.0 with the following optimization and standardization flags: [-O3 -funroll-loops -march=native -std="c++11"]. Modules were used from the NGraph C++ library and Network Tookit [26], as well as optimized C++ implementations of algorithms noted in the paper.

Timing results are reported in milliseconds (msec) due to the effectiveness of the optimized modules. As these timing results are rather small, each operation was repeated and their average duration is reported. Only the larger networks and largest *k* value are reported, as the smaller cases simply ran too fast to be captured by the resolution of the system clock.

**Table 8:** Timing analysis of largest networks: computation time to find *F*_10_($\hat{A}$) via semi-greedy algorithm, compared to time required to find 𝕄_1_*_,_*_000_[*F*_10_()], expressed as the ratio of increased speed.

Network	Semi-greedy (ms)	speed increase
NetScience	14.4	5.38 × 10^4^
*C. elegans*	12.5	2.98 × 10^3^
Arenas email	51.0	1.23 × 10^4^
FAA air traffic	24.7	1.90 × 10^4^
Human protein	14.9	2.18 × 10^5^
ca-GrQc	40.4	3.90 ×10^5^
ca-HepTh	71.1	1.04 × 10^6^
ca-HepPh	156.8	9.39 × 10^5^

The results denote the performance of the semi-greedy algorithm (Fig. 2) with the target set size *k* = 10, and the number of hubs to explore *h* = 100. The main point to illustrate is that these methods yield rather good near-optimal approximations on graphs having thousands of vertices and edges in a **fraction of a second**. (In fact, the time required to solve the problem is often shorter than the time required to read the network data from disk.) Speed and efficiency are critical, as these network problems may be embedded in a tight loop within larger time-dependent applications.

## 8 Conclusion

In Ref. [9], the authors posed the problem of finding a subset of target vertices of fixed cardinality that minimized the sum of the expected time taken by random walkers in a network to arrive at the set for the first time. The solution to this problem identifies a set that is an optimal influencer for a consensus process as modeled by a random walk. This paper presents a methodology for identifying optimal influencers in graphs with real world applications. Because the size of such networks can range from hundreds to millions of nodes, brute force evaluation of expected arrival times is unfeasible. Indeed, a simpler variant of this problem, where the average random-walks of *F*(A) are replaced with a straightforward graph distance, results in a case of the **k-median problem** in a metric space. This problem has been shown to be computationally difficult [27], belonging to a class of non-deterministic polynomial-time hard (**NP-hard)** problems which have eluded efficient solutions. Beginning with [9], research has focused on methods that exploit the supermodularity property of the arrival times [16, 28, 29] but they have not been applied to the largest networks, such as those we consider here, and may not be feasible in these cases.

To meet this challenge, we developed heuristics that exploit the heavy-tailed degree distribution found in many large graphs of importance in real-world applications. The algorithms we discuss in Sec. 5 hinge on the construction of candidate sets in which the vertices are network hubs. These hubs are selected so that neighborhoods (*p*th order neighborhoods) of distinct hubs have minimal overlap, and remote areas of the network are more likely to be covered. In this setting our algorithms and heuristics rapidly produce a relatively small set of candidate sets.

The offered solution is then obtained by evaluating a Monte Carlo approximation of the expected arrival times to the candidate sets. The implementation performed on larger graphs (algorithm in Fig. 2) took fractions of seconds to find $\hat{A}$, in our experiments. Evaluating the accuracy of these methods is difficult, but in Sec. 6, we took the first steps toward this goal. We used existing results on stochastic optimization and statistics to establish the validity of the Monte Carlo approximation of expected arrival times.

Because of the practical difficulty of directly assessing the accuracy of the offered solution, we propose instead for future work, to estimate *c*, using the *η*th quantile of the cumulative distribution of {*F*(*A*)}_|_*_A_*_|=_*_K_* values for small *η*. Using independent replication of the algorithm, and Chebyshev's inequality (see Appendix) the estimate can be shown to be reliable with high probability given a sufficient number of repetitions. The value of *c* provides an upper bound to both the actual solution and the offered solution of our problem.

We also determined a correlation between the potential increase in speed to near-optimal sets, 𝔼[*F_k_*()]*/F*($\hat{A}$), and the ratio of maximum to average degree of the network (Δ*/*(*d*)). This can be used a guide to help determine how much to expect from near-optimal solutions.

For smaller graphs, it is possible to compute these optimal sets exactly through exhaustive search, and we did so here to validate the expectation that the heuristics do produce reasonable results. In about a third of the cases, the algorithms find the true optimal *F*(*A*) value, while at worst still remaining within a 1 - 1*/e* bound. For larger graphs (say *V* ≳ 100) computing the exact optimal is impractical, so we estimated it by designating the top 0.1% ranking of 1,000 samples and comparing it to the heuristic solution, expressed as 𝕄_1_*_,_*_000_[*F_k_*()]*/F*($\hat{A}$). In our experiments, this ratio was greater or equal to 1 in 98% of the cases.

Finally, we demonstrated that the heuristics are computationally efficient. The largest network in our test cases, *ca-HepPh*, with *k* = 10 target sets, took 41 h of computation to find candidate sets in the top 0.1% ranking of 1,000 samples, while the heuristics found significantly better solutions in 0.16 s. This near million-fold increase in speed brings problems that were computationally cumbersome into the nearly instantaneous regime and opens up new possibilities for researchers and application specialists.

## 9 Appendix A: Proof of Equation (12)

In this section, we will present a proof of the formula that reveals the relationship between the rate of convergence to network consensus in a network containing a subset *A* of leader nodes and the first hitting (or arrival) time of a random walker in the network graph that begins at a node outside of *A*.

**Lemma 1.** *Let* (*V,* ℙ) *be the sample space and probability measure of an irreducible Markov chain with transition matrix P and suppose A* ⊂ V is a non-empty subset. If x_n_ is the vector at time n of the consensus process in Eq. (10), with limiting vector x*,then

||x* - *x_n_*||_1_ = ℙ[*T_A_ > n*] (26)

*Proof.* From Eq. (11) we know that ||x* - *x_n_*||_1_ = *c*||$P_{A}^{n}$**1**||_1_. Now the *i*th component of $P_{A}^{n}$**1** is ∑ *_j_*_∈_*_A_c P^n^*(*i, j*) = *P_i_*[*T_A_ > n*], the probability that starting at *i*, the random walker has not arrived at *A* after *n* steps. Thus to compute the *l*_1_ norm we must have,

${\left\|x*-x_{n}\right\|}_{1}=\sum_{i\in A^{c}}P_{i}\left[T_{A}>n\right]=\mathbb{P}\left[T_{A}>n\right]$

## 10 Appendix B: Statistical Approach to Validating the Heuristic Methods

We will use the notation introduced in Sec. 6 to give a brief description of an approach for estimating the value *c* and associated confidence intervals. In this analysis we assume the sets that appear in the latter stages of the heuristic have *F* values that come from the lower tail of the distribution. Let *η* be the quantile level and *n* the number of sets selected to be in the final random simulation for estimating *F*($\hat{A}$). Given *η*,we choose *n* = $\left\lfloor \eta \cdot \left(\begin{array}{l} \left|V\right|\\ k \end{array}\right)\right\rfloor$. This last collection of sets contains the offered solution corresponding to the smallest value of the approximate *F* value. The largest approximate *F* value is $\hat{c}$, an estimate of *c*. Note that $\hat{c}$ depends on *η*. In addition we make some additional assumptions:

• The tail of the distribution of F values decays rapidly enough so that a second moment is well defined.
• $\hat{c}$ is a random variable with mean *c*. In addition to random fluctuations due to errors arising from the Monte Carlo approximation of *F*, there are also sampling errors that affect the tail distribution and thus the value of *η*.

Suppose we estimate *c* by averaging the values $\hat{c}$ obtained by *T* multiple independent repetitions of the algorithm. We assume these are done so that the random variables in different trials have the same distribution but are statistically independent. If *X_T_* =$\sum_{i=1}^{T}{\hat{c}}_{i}$, where ${\hat{c}}_{i}$ is calculated in the *i*th trial, then for any *ε >* 0, Chebyshev's inequality implies that,

$P\left\{\left|X_{T}/T-c\right|>\varepsilon \right\}<\frac{Var\left(X_{T}\right)}{\varepsilon^{2}}=\frac{Var\left(c\right)}{T^{2}\varepsilon^{2}}$(27)

The probability measure *P* refers to the sample space of Monte Carlo simulations. Here the notation *Var*(*X*) denotes the variance of *X* . The quantity *Var*(*c*) can be estimated using the sample variance of the trial values. The right-hand side of the inequality in Eq. (27) shows that *c* lies in an interval around $\frac{X_{T}}{T}$ of length 2*ε* with degree of confidence given by probability $1-\frac{V(c)}{T\varepsilon^{2}}$ . Note that a considerable amount of computation may be needed to attain a high degree of precision in this sense. Further research is needed to determine if this method is feasible for graphs where the algorithm is very fast.
